# Turner syndrome and growth hormone therapy: a review on growth response

**DOI:** 10.1186/1687-9856-2015-S1-P37

**Published:** 2015-04-28

**Authors:** Nalini M Selveindran, Janet YH Hong, Arini Muhd Idris, SL Teoh, Karen SW Leong, Fuziah M Zain

**Affiliations:** 1 Paediatric Department, Hospital Putrajaya, Presint 7, Putrajaya, Wilayah Persekutuan, Malaysia

## Aims

Turner syndrome (TS) is a sex-chromosome abnormality in females resulting from partial or complete absence of one of the X chromosomes [1]. One of the main features of TS is short stature. Final height in short girls with TS improves with growth hormone treatment [2]. The aim of this study was to assess the effectiveness of growth hormone therapy in a cohort of Malaysian patients with TS.

## Methods

Data from the electronic medical records of 20 patients with TS treated with growth hormone in Putrajaya Hospital, Malaysia from the year 2005 to 2014 was analysed.

## Results

The mean age of initiation of therapy was 11.5 ± 3.4 and 55.0% were started after the age of 12 years. The mean height standard deviation score (SDS) increased from -3.84 (±0.94) SD at study entry to -3.47 (±0.97) SD at the end of the first year. This improvement was seen with subsequent year of treatment, though the degree of change in height SDS reduced with time. Age of initiation of therapy had a bearing on treatment response as those who had received growth hormone at an earlier age experienced better growth response. 75.0% of patients who achieved final height were able to achieve the final height within the target height. Of the patients who achieved final height, mean age of puberty was 16 ± 1.5 years and mean height SDS at onset of puberty was -3.3SD. There were no reported adverse events.

## Conclusion

Growth hormone therapy improved growth profile of Malaysian children with TS and the importance of early initiation of therapy is demonstrated in this study.
